# Recurrence in unicentric castleman’s disease postoperatively: a case report and literature review

**DOI:** 10.1186/s12893-017-0334-7

**Published:** 2018-01-04

**Authors:** Na Ren, Lei Ding, Erna Jia, Jinru Xue

**Affiliations:** 1 Department of Thoracic surgery, China-Japan Union Hospital of Jilin University, Number 126, Xiantai street, Changchun, 130033 China; 2 Department of Radiology, China-Japan Union Hospital of Jilin University, Changchun, 130033 China; 3 Department of Gastroenterology, China-Japan Union Hospital of Jilin University, Changchun, 130033 China

**Keywords:** Unicentric castleman’s disease, Hyaline vascular type, Sugery, Recurrence, Postoperative

## Abstract

**Background:**

Our case describe a rare recurrence case of Unicentric Castleman’s disease (UCD) with hyaline vascular type 14 years after surgery.

**Case presentation:**

A 35-year-old Chinese female was admitted to hospital with one and half months history chest distress and chest pain. Patient reports a history of thoracic operation for mediastinal mass 14 years ago, and it was diagnosed UCD with hyaline vascular type after postoperative pathological examination. At this time, the imaging examination showed a mediastinal mass once again. Combining the medical history, postoperative microscopically examination and immunoperoxidase staining, patient was again diagnosed UCD with hyaline vascular type again. The hyaline vascular type is the most common type and usually presents as a UCD. Most patients with UCD have no clinical symptoms. The diagnosis of UCD is generally achieved with histological and immunohidtochemical findings postoperatively. Currently, the standard treatment of UCD is the complete surgical resection, with almost no relapse postoperative. The recurrence in UCD with hyaline vascular type postoperative have not previously been reported. Therefore, herein we describe a recurrence case of UCD with hyaline vascular type 14 years after surgery.

**Conclusion:**

Our case is the first case which reports the relapse of UCD with hyaline vascular type after completely surgery. It indicates that long term follow-up is necessary for patient who is diagnosed UCD after surgery.

## Background

Castleman’s disease (CD) is a rare atypical lymphoproliferative disorder that can be easily misdiagnosed. Benjamin castleman [1] first described a cohort of patients with solitary hyperplastic mediastinal lymph nodes which demonstrated small, hyalinized follicles and interfollicular vascular proliferation on histopathology. Then was originally identified by Benjamin castleman in the year 1954. The overall incidence of the disease is estimated to be less than 1/100,000 [2]. CD generally occurs in young adults [3] and has no gender predilection [4].

CD is an uncommon form of disorders characterized by proliferation of morphologically benign lymph nodes hyperplasia. Clinically, it may present as either unicentric (localized, UCD) or multicentric (systemic, MCD). Keller [5] classified three CD histological subtypes: hyaline vascular type (80%–90%), plasma cells type (10%), and mixed type (2%). The hyaline vascular type is the most common type and usually presents as a unicentric form, as a mass confined to a single lymph node or a group of lymph nodes [6]. Most patients have no clinical symptoms and typical imaging features. Due to the risk of massive hemorrhage during biopsy by needle aspiration, the diagnosis of UCD is generally achieved with histological and immunohidtochemical findings after surgical resection [7]. UCD generally involves focal lesions and has a better prognosis for long-term survival after surgical treatment of the lesion resection. Complete surgical resection is the standard treatment of UCD, which is a curative method in 95% of localized form patients, and almost there are no relapse after surgery [8].

Recently, very few relapse case of UCD with hyaline vascular type was reported. Thus we report here an unusual case of relapse UCD with hyaline vascular type after 14 years of surgical resection.

## Case report

**Fig. 1:**
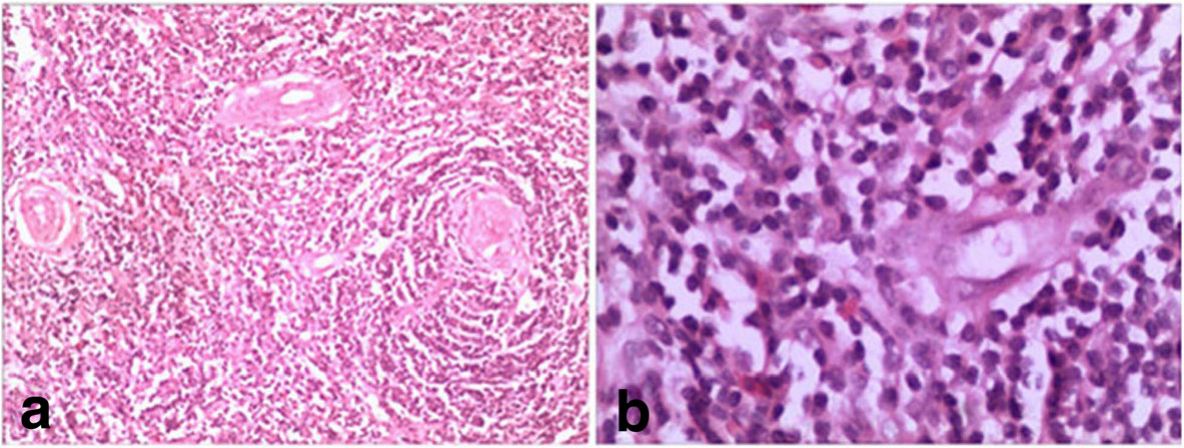
**a** histology of the tissue showed proliferation of lymphoid follicles (H&E, 100X); (**b**), lymph node with lymphoid cells in an “onion skin” pattern with a hyaline center (H&E, 100X)

A 35-year-old Chinese female was presented with chest distress and chest pain for one and a half months from November 2014. She had no obvious dyspnea, pant and cardiopalmus. Her chest pain aggravated progressively without any obvious exacerbation and alleviation factors. She had no chronic lung disease or cardiac disease history. And no fever, night swears, or weight loss was observed. But she had a history of thoracic operation 14 years ago because of a mediastinal mass, which diagnosed UCD with postoperative pathological examination. The postoperative pathologic result was consistent with hyaline vascular Castleman’s disease (Fig. 1a and b). The mass was located in the right mediastinum, above the precava and vicinity of the esophagus, measuring 6.0 cm × 5.0 cm ×3.0 cm mass in size. It is lobulated in shape, with a large amount of surface vessel.

The Physical examination revealed no abnormal results. The patient’s biochemical profile, full blood count, erythrocyte sedimentation rate, and tumor marker test were normal. The result of the human immunodeficiency virus (HIV) screening test was also negative.

**Fig. 2:**
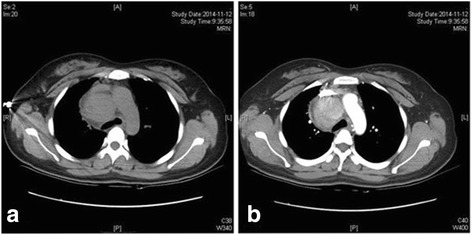
**a** the non-enhanced phase revealed a homogeneous, fleshy, noninvasive, solitary mass; (**b):** the arterial phase revealed evident contrast heterogeneous enhancement in the mass

Imaging revealed a homogeneous, noninvasive, large, solitary mass in the mediastinum at the local hospital. Then the patient was referred to our hospital, received chest contrast-enhanced computed tomography (CT) scans, which confirmed a mass (6.0 cm × 3.8 cm) at the right mediastinum. The non-enhanced phase revealed a homogeneous, fleshy, noninvasive, solitary mass, the value of the CT was 16HU-42HU (Fig. 2a), and evident contrast heterogeneous enhancement was observed in the mass during the arterial phase (Fig. 2b).

The patient had a history of thoracotomy 14 years ago because of a mediastinal mass. Considering a great possibility of the pleural adhesion after surgery and the location of the tumor, the thoracoscopic approach was not suitable. The patient underwent thoracotomy and mass resection from the right anterrolateral incision. Intraoperatively, the right mediastinum tumor was seen, the tumor (before the trachea, behind the superior vena cava, over the root of vena cava, under the brachiocephalic vein) was 5 cm × 5 cm × 6 cm in size, firm, a rich and large vascularity in the surface.

**Fig. 3:**
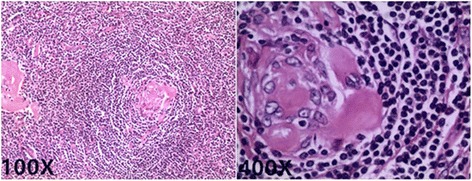
Histology of the tissue revealed proliferation of lymphoid follicles, Lymph node with lymphoid cells in an “onion skin” pattern with a hyaline center (H&E, 100X; 400X)

Postoperative microscopically examination (Fig. 3) revealed a mass consisting of lymphoid tissue with a large number of vascular invasion, and laminated mantle zones with concentric rings of small lmphyocytes surrounding atrophic germinal centers, as an “onion skin”. A hyalinlied interstitium and numerous vascular structures was observed between the follicles.

**Fig. 4:**
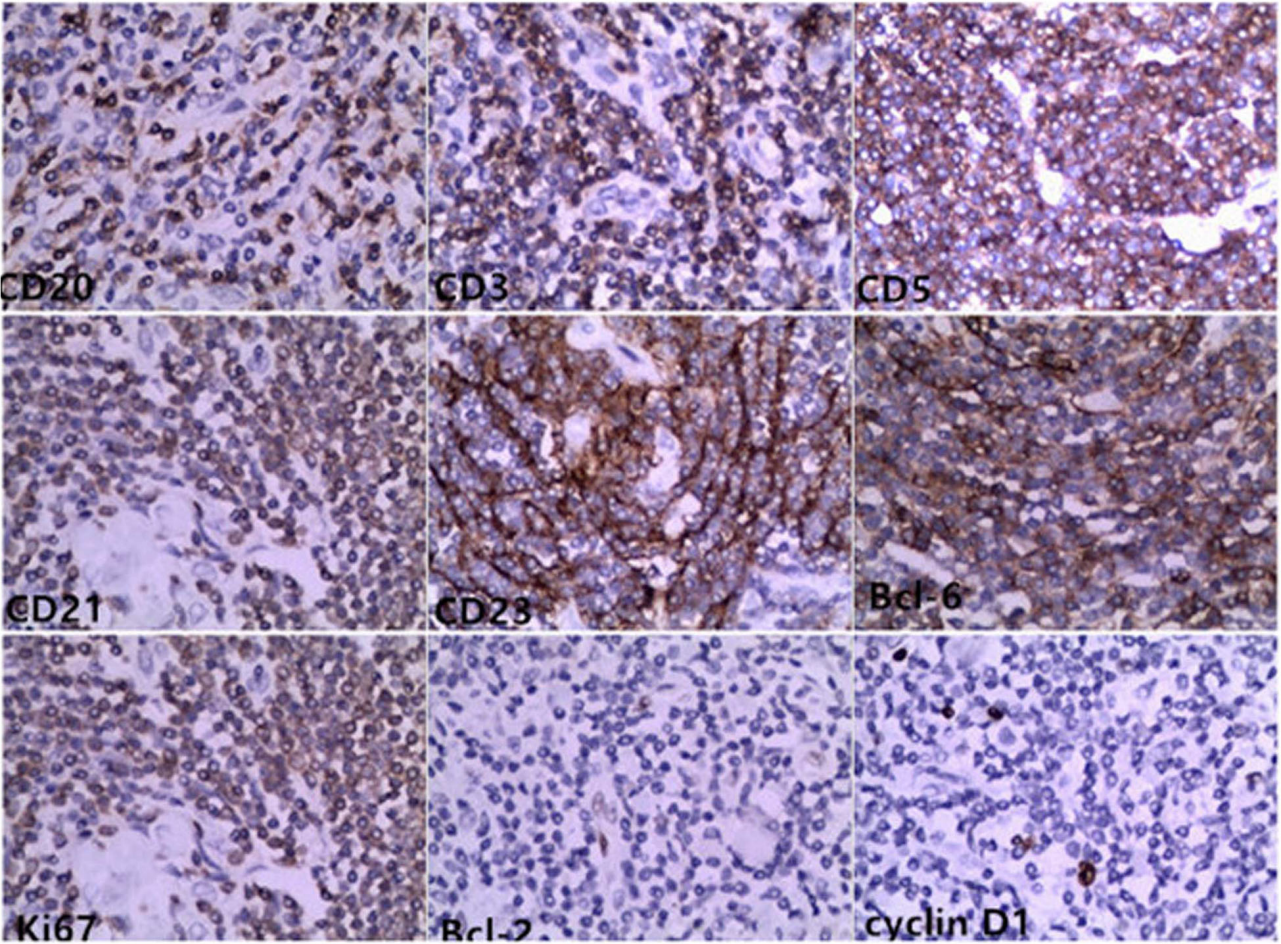
Immunohistochemical staining for CD20, CD3, CD5, CD21, CD23, BCL-6, Ki67 positivity and BCL-2, cyclin D1 negativity demonstrated follicular hyperplasia (H&E, 4 00X)

Immunoperoxidase staining (Fig. 4) showed CD20 and CD79a reactivity in the B lymphocytes population, CD3 and CD5 reactivity in the T lymphocytes population, CD21 and CD23 reactivity in the follicular dendritic cells. BCL-6 and Ki67 markers were detacted positive. Negativity for CD10, BCL-2, and cyclin D1 markers were also detacted.

The patient recovered well postoperatively and discharged 10 days after the operation. His chest distress and chest pain had completely alleviated. During the following 2 years the patient had no progression or recurrence of the disease.

## Discussion and conclusions

Unicentric Castleman’s disease (UCD)is a localized form of hypervascular lymphoid hyperplasia. It accounts for most of the cases and presents as a mass located in the thorax (30%), the neck (23%), the abdomen (20%), or the retroperitoneum (17%) and rarely affects the axillary region (5%), the groin area (3%), or pelvic region (2%) [9].Aother study report the most common site of involvement of UCD is the mediastinum (70%) [5], but it can occur wherever lymph node is present [10], for example [11] pancreas, liver, kidney and neurological system. UCD of the mediastinum, wihch is also known as angiofollicular giant lymph node hyperplasia, is benign lymph node hyperplasia. Clinically, the hyaline vascular type comprises up to 90% of cases and the plasma cell type comprises approximately 10–20% of cases in UCD [7].

The etiology and pathogenesis of CD is remain uncertain and has been described in association with a reactive lymphoid hyperplasia initiated by viral infections or a development growth disturbance of the lymphoid tissue [12]. Current hypotheses speculate chronic low grade inflammation, hamartomatous process, immnodefeicent state or autoimmunity as potential etiology [3]. Certain researches [13] have proposed that CD is associated with human herpes virus 8 (HHV-8) and HIV infection. Moreover, a dysregulation in IL-6 overproduction is thought to play an important role in lymphoid hyperplasia and possibly the pathogenesis of CD [14].

Clinical symptoms of UCD are closely correlated with the pathological type. It mainly involves lymph node enlargements only a single site, and 90% of UCD patients are usually asymptomatic, have no indolent, slow progressive course and a rare discovery on radiographs. The clinical, radiological, or cytological typical features are relevant, confirming the correct diagnosis is difficult before the surgery. In our case, the chest contrast-enhanced computed tomography (CT) scans revealed a mediastinal mass. The non-enhanced phase revealed a homogeneous, fleshy, noninvasive, solitary mass and evident contrast heterogeneous enhancement was observed during the arterial phase. According to the medical history, clinical presentation, and the findings of chest contrast-enhanced computed tomography imaging, this case might not be diagnosed as the recurrence of UCD.

The definite diagnosis of UCD is exclusively based on histological and immunohistochemical finding after resection [7]. Microscopically, the Characteristic manifestations [15, 16] present a typical lymph node background, a capsule, and a classic large number of lymphoid follicles. Parts of these follicles present atrophic germinal centers, abundant hyaline vessels, and surrounding with small lymphocytes in the peripheral wide zones, the germinal centers have an “onion skin” appearance, which is the typical feature of UCD, as well as the hyaline vessels across the peripheral wide zones into germinal centers. Immunohistochemically [16], CD20 was detacted positive staining on the B lymphocytes population, CD3 and CD5 were detacted positive staining in the T lymphocytes population, CD10, BCL-6 and Ki67 were detacted positive staining in the germinal center population, and CD21 and CD23 were detacted positive staining in the follicular dendritic cells. Negativity for HHV8, CD56, TDT, BCL-2, and cyclin D1 markers were also detacted. In our case, postoperative histology of the tissue revealed proliferation of lymphoid follicles, Lymph node with lymphoid cells in an “onion skin” pattern with a hyaline center; Immunohistochemical staining for CD20, CD79a, CD3, CD5, CD21, CD23, BCL-6, Ki67 positivity and CD10, BCL-2, cyclin D1 negativity demonstrated follicular hyperplasia. These observations all indicated the diagnosis of UCD. Evermore, according to the history of the patient before 14 years, the conditions of the mass observed intraoperatively and the postoperative histology of the tissue, all conformed the diagnosis. As the limits of the techniques of local hospital 14 years ago, the Immunohistochemical examination of the mass had not been tested. Horever, based on all inspection findings, it could be definitely diagnosed as UCD with hyaline vascular type.

In UCD, surgical resection of the mass is a standardized and preferred treatment protocol, the curative ratio can reach 95%. Some researches report surgical treatment can achieve a cure rate of approximately 100%, either with vascular type or plasma cells type [9]. Recently, Radical radiotherapy [17, 18] is used to those who unable to undergo surgery because of medical disorders, or who refuse surgery. Postoperative radiotherapy for UCD is recommended because of the possibility of relapse after partial excision [17, 18]. At present, there is no standard protocol for predicting the prognosis and effectively managing UCD, and there is no recrudescent case of UCD with hyaline vascular type postoperatively reported. Then our case is the first report of recrudescent UCD with hyaline vascular type after 14 years postoperatively.

UCD is a rare disease and difficult to diagnose. We emphasize that surgery remains as the mainly treatment means. So far, a number of studies have reported that the surgical resection of UCD can achieve a cure rate of approximately 100%, and there is no relapse postoperatively during the reported followup [6, 9, 11]. However, in our case report, the patient with chest distress and chest pain comes to our hospital and is diagnosed again as UCD 14 years after thoracotomy. It indicates that the relapse of UCD after surgery is possible. Our case is the first case which reports the relapse of UCD with hyaline vascular type after completely surgery. It indicates that long term follow-up is necessary for patient who is diagnosed UCD after surgery.

## Abbreviations

CD: Castleman’s disease
MCD: Multicentric Castleman’s disease
UCD: Unicentric Castleman’s disease
